# Refining genotype–phenotype correlation in Alström syndrome through study of primary human fibroblasts

**DOI:** 10.1002/mgg3.296

**Published:** 2017-05-15

**Authors:** Jian‐Hua Chen, Tarekegn Geberhiwot, Timothy G. Barrett, Richard Paisey, Robert K. Semple

**Affiliations:** ^1^ Wellcome Trust‐MRC Institute of Metabolic Science University of Cambridge Cambridge UK; ^2^ The National Institute for Health Research Cambridge Biomedical Research Centre Cambridge UK; ^3^ University Hospital of Birmingham Birmingham UK; ^4^ University of Birmingham Birmingham UK; ^5^ South Devon Healthcare NHS Foundation Trust Torbay Hospital Torquay UK

**Keywords:** ALMS1, Alström syndrome, cilia, gene expression, genotype–phenotype correlation, nonsense‐mediated decay

## Abstract

**Background:**

Alström syndrome (AS), featuring retinal dystrophy, neuronal deafness, cardiomyopathy, metabolic syndrome, and diffuse fibrosis, is caused by biallelic mutations in the centrosomal protein ALMS1. Genotype–phenotype correlation has been suggested without assessment of ALMS1 expression.

**Methods:**

ALMS1 expression (real‐time PCR and immunocytochemistry) and cilia formation (immunocytochemistry) were assessed in fibroblasts from deeply phenotyped volunteers diagnosed with AS recruited from a dedicated AS Service. Exome sequencing was used in two participants without convincing biallelic *ALMS1* mutations, and BBS2 (Bardet–Biedl syndrome 2) protein expression was assessed in one patient with biallelic *BBS2* mutations. Hedgehog‐induced GLI1 expression and PDGFA signaling was assessed using quantitative real‐time PCR, immunoblotting, or immunostaining of fixed cells after stimulation.

**Results:**

In 16 of the patient cell lines examined, ALMS1 protein was undetectable (14 with biallelic loss‐of‐function (LoF) mutations), and in two, ALMS1 staining was equivocal (one with biallelic LoF mutations). In five lines, ALMS1 expression was normal using at least one fixation method (one with biallelic LoF mutations). These differences were not accounted for by major differences in *ALMS1* mRNA expression. Exome sequencing of two participants with normal ALMS1 expression identified biallelic LoF *BBS2* mutations in one. No second, known ciliopathy mutation was found in the other patient, who had one LoF ALMS1 mutation. Phenotypes were milder or atypical in participants with preserved ALMS1 immunostaining, even when two with likely alternative genetic diagnoses were excluded. All cells studied developed normal cilia, *ALMS1* and *BBS2* mutant cells showed normal Hedgehog‐induced upregulation of GLI1 expression, and PDGFA signaling was normal in ALMS1‐deficient cells.

**Conclusion:**

Milder or atypical presentations of AS should prompt genetic evaluation for alternative, clinically overlapping ciliopathies. A subgroup of patients with *bona fide* ALMS1 defects have milder phenotypes due to residual ALMS1 expression, which may be more important than mutation site.

## Introduction

Alström syndrome (AS; OMIM #203800) is a rare (c. 1 per million) autosomal recessive condition characterized by childhood onset retinal dystrophy, neuronal hearing loss, obesity and insulin‐resistant diabetes (Marshall et al. 2007a). Since its original description infantile and adult cardiomyopathy, renal and hepatic dysfunction have also been recognized as important clinical features of the syndrome. It is known to be caused by biallelic mutations in the *ALMS1* gene (Collin et al. 2002; Hearn et al. 2002); however, although the syndrome was first described in 1959, and although the genetic basis has been known for more than a decade, the mechanisms linking the genetic defect to organ dysfunction are largely unknown.

The *ALMS1* gene encodes a very large, ubiquitously expressed protein that is associated with the centrosome and the basal body of the primary cilium (Hearn et al. 2005). This, allied to the pattern of organ dysfunction seen, has led to Alström syndrome being classified as one of the growing number of “ciliopathies”, caused by defects in primary cilium formation or function (Girard and Petrovsky 2011). Primary cilia are evolutionarily conserved, membrane‐bound, microtubular projections emanating from the cell surface and present on virtually all cell types in the human body (Kim and Dynlacht 2013). They function as signaling “antennae”, having dense expression of receptors and channels on the ciliary membrane to sense, integrate, and transduce extracellular cues such as growth factors, hormones, odorants, and developmental morphogens. Cilia play an indispensable role in tissue development (Singla and Reiter 2006; Gerdes et al. 2009), chemosensation, thermosensation, mechanosensation, osmosensation, and photoreception (Lancaster and Gleeson 2009; Oh and Katsanis 2012). It is therefore not surprising that ciliary defects affect multiple organs and cause a wide range of diseases (Fliegauf et al. 2007; Hildebrandt et al. 2011).

Despite the strong circumstantial case that Alström syndrome is a *bona fide* ciliopathy, direct evidence for ciliary dysfunction in Alström syndrome is sparse. Cells and tissues from affected patients have morphologically grossly normal primary cilia (Hearn et al. 2005), although subtle defects in stereocilia (Jagger et al. 2011), renal tubular cells (Li et al. 2007), and hypothalamic neurones (Heydet et al. 2013) have been described in murine models of Alström syndrome. It has been speculated that loss of ALMS1 leads to functional rather than anatomical defects in cilia, compromising, for example, vesicle transport from the Golgi apparatus to the cilium and/or intraflagellar transport (Hearn et al. 2005; Girard and Petrovsky 2011). It remains possible that ALMS1 plays other roles in cells unrelated to primary cilia, however, and that it is loss of such functions that relates to the tissue pathology of Alström Syndrome.

Previous genetic studies have raised the tentative possibility that there is discernible genotype–phenotype correlation within Alström syndrome, with associations reported between mutations in exon 16 and early retinal disease, urological dysfunction, cardiomyopathy, and diabetes, and between mutations in exon 8 and relative protection from renal disease (Marshall et al. 2007b). However these studies did not assess whether any ALMS1 protein product was produced from the mutant alleles identified. Moreover although most of the mutations in the patients reported were nonsense or frameshift mutations, eight missense mutations of uncertain pathogenicity were also included (Marshall et al. 2007b).

We have now studied 23 primary dermal fibroblast lines from patients with a clinical diagnosis of Alström syndrome, all of whom had undergone mutational analysis of the *ALMS1* gene. We examined ALMS1 protein expression and ciliogenesis in an attempt to re‐examine the possibility of genotype–phenotype correlation in Alström syndrome, and, in view of the role of the primary cilium as an indispensable cellular signaling organelle (Christensen et al. 2012; Briscoe and Therond 2013), we assessed Hedgehog and PDGFA (Platelet‐derived growth factor subunit A) signaling in a selection of affected cells.

## Materials and Methods

### Clinical assessment and ethical compliance

All participants underwent clinical assessment in dedicated Alström syndrome clinics at Torbay Hospital, Birmingham Children's Hospital or Queen Elizabeth Hospital Birmingham, set up in collaboration with Alström Syndrome UK, with additional clinical and cellular phenotyping studies undertaken as part of a research study approved by the UK National Research Ethics Committee. All volunteers provided written informed consent, and the study was conducted in accordance with the principles of the declaration of Helsinki. Diagnostic testing of the *ALMS1* (NM_015120.4) gene was performed in accredited diagnostic laboratories, with all mutations now numbered with reference to ALMS1 canonical transcript ENST00000613296.4.

### Skin biopsies, establishment and maintenance of primary dermal fibroblasts

Punch skin biopsies were taken from the flank, before disaggregation and culture in Dulbecco's modified Eagle Medium (DMEM; D6546, Sigma‐Aldrich, Haverhill, UK) supplemented with 10% fetal bovine serum (SV30180.03, GE Healthcare Bioscience, Buckinghamshire, UK), 2 mm L‐glutamine (G7513, Sigma‐Aldrich, Haverhill, UK), and 1% penicillin–streptomycin (P0781, Sigma‐Aldrich, Haverhill, UK) in a humidified incubator (37°C, 5% CO_2_). Cells were passaged once weekly with 1:4 splitting.

### Immunofluorescence analysis of ALMS1 expression and ciliogenesis

Fibroblasts prepared on coverslips were serum starved for 24 h to induce cilia formation. Cells were then fixed using one of two approaches as indicated. In the first, they were fixed in 4% paraformaldehyde in PBS for 10 min followed by one wash with TBS [50 mm Tris–HCl (pH7.4), 150 mm NaCl], permeabilization in 0.2% Triton X‐100 in PBS for 5 min, three washes with TBS and quenching in fresh 0.1% sodium borohydride in TBS for 5 min. Alternatively, cells on coverslips were fixed with 100% methanol (prechilled to −20°C) for 10 min followed by three washes with TBS and quenching in fresh 0.1% sodium borohydride in TBS for 5 min. Coverslips were blocked with blocking buffer (10% horse serum, 1% BSA, 0.02% NaN_3_, 1× PBS) for 1 h, washed with TBS, and incubated with anti‐ALMS1 (ab84892, abcam, Cambridge, UK) and anti‐acetylated tubulin (T7451, Sigma‐Aldrich, Haverhill, UK), anti‐γ‐tubulin (T5326, clone GTU‐88, Sigma‐Aldrich, Haverhill, UK), anti‐Smoothened (ab72130, abcam, Cambridge, UK), anti‐PDGFR‐α (sc‐338, Santa Cruz, via Insight Biotechnology, Wembley, UK), or anti‐phosphor‐MEK1/2 (#9121, Cell Signaling Technologies, Leiden, The Netherlands) in 1% BSA in TBS overnight at 4°C. After washing, the cells were incubated with 1:1000 dilution of Alexa Fluor^®^ 488 goat anti‐mouse IgG (A11001, Invitrogen, via Thermo Fisher Scientific, Hemel Hempstead, UK) and Alexa Fluor^®^ 555 goat anti‐rabbit IgG (A21430, Invitrogen, via Thermo Fisher Scientific, Hemel Hempstead, UK) for 45 min at room temperature in the dark, washed with TBS, mounted on glass slides using the ProLong Gold Antifade Reagent with DAPI (P36931, Invitrogen, via Thermo Fisher Scientific, Hemel Hempstead, UK) and inspected with a Zeiss LSM510 Meta (Carl Zeiss Microscopy) or Leica TCS SP8 confocal laser scanning microscope (Leica Microsystems, Milton Keynes, UK).

### Hedgehog and PDGFA signaling pathway assay

Fibroblasts were serum starved for 24 h in DMEM containing 0.5% BSA (A8412, Sigma‐Aldrich, Haverhill, UK), 2 mm L‐glutamine, and 1% penicillin‐streptomycin. Cells were then treated with 1 μm SAG (Smoothened agonist, 566661, Calbiochem, San Diego, USA), 0.25 μg/mL SHH (1845‐SH‐025, R&D Systems, Abingdon, UK), or 50 ng/mL PDGF‐AA (W1800950002, BIOCHROM AG, Berlin, Germany) in serum‐free DMEM for 24 h. Cells were either fixed for immunofluorescence analysis as above or harvested for western or qRT‐PCR analysis.

### Western blot analysis

Cells were washed with ice‐cold PBS and harvested in M‐PER Mammalian Protein Extraction Reagent (78503, Thermo Fisher Scientific, Hemel Hempstead, UK) containing freshly added protease inhibitor mini complete cocktail (11 836 153 001, Roche, via Sigma‐Aldrich, Haverhill, UK). Lysates were mixed with equal volume of 2× Laemmli Sample Buffer (1610737, Bio‐Rad Laboratories, Watford, Hertfordshire, UK) and denatured at 100°C before being resolved by SDS‐PAGE and transferred to PVDF membranes using the iBlot system (Invitrogen, via Thermo Fisher Scientific, Hemel Hempstead, UK). Blots were blocked in TBST (50 mm Tris‐HCl, pH7.6, 150 mm NaCl, 0.1% Tween‐20) containing 5% milk or BAS and probed overnight at 4°C with the following antibodies: anti‐BBS2 (11188‐2‐AP, proteintech, Manchester, UK) anti‐GLI1 (#3538, Cell Signaling Technologies, Leiden, The Netherlands), anti‐GLI2 (#2585, Cell Signaling Technologies, Leiden, The Netherlands), anti‐GLI3 (MABS275, Millipore, Watford, UK), anti‐PDGFRA (sc‐338, Santa Cruz, via Insight Biotechnology, Wembley, UK), anti‐MEK1/2 (#9122, Cell Signaling Technologies, Leiden, The Netherlands), anti‐phosphor‐MEK1/2 (#9121, Cell Signaling Technologies, Leiden, The Netherlands), or anti‐phospho‐AKT (#5102, Cell Signaling Technologies, Leiden, The Netherlands). Horseradish peroxidase‐conjugated secondary antibodies were used followed by Immobilon Western Chemiluminescent HRP Substrate (WBKLS0500, Millipore, Watford, UK).

### mRNA quantification

Total cellular RNA was prepared using RNeasy Mini Kits (Qiagen, Manchester, UK) with a DNase digestion step included. First strand cDNA was reverse‐transcribed from 400 ng of total RNA using an ImProm‐II Reverse Transcription System (A3800, Promega, Southampton, UK) with random hexamer primers.

Quantitative real‐time PCR was carried out using an ABI PRISM 7900 Sequence Detection System (Applied Biosytems, via Thermo Fisher Scientific, Hemel Hempstead, UK) with a SYBR Green PCR Master Mix (4309155, Applied Biosystems) and gene‐specific primers. Primers were custom‐designed and synthesized by Sigma: GLI1 forward primer (5′ to 3′) GGCTGCAGTAAAGCCTTCAG, GLI1 reverse primer (5′ to 3′) GCAGCCAGGGAGCTTACATA, HPRT1 forward primer AGTTCTGTGGCCATCTGCTT, and HPRT1 reverse primer TAGGAATGCAGCAACTGACA. For every gene analyzed dissociation curve analysis was undertaken. Samples were run in duplicate, and standard curves were constructed using serially diluted pooled cDNA. *HPRT1* was used as an endogenous loading control after verification that its expression was equal between treated and untreated dermal fibroblasts.

### DNA sequencing

Exome sequencing was undertaken by Oxford Genome Technologies. In brief, DNA samples were prepared according to Agilent's SureSelect Protocol Version 1.2 with enrichment carried out according to Agilent SureSelect protocols, and sequencing undertaken on the Illumina HiSeq2000 platform using TruSeq v3 chemistry. Read files (Fastq) were generated via the manufacturer's proprietary software, reads were mapped to the hg19/b37 build of the human genome using the Burrows‐Wheeler Aligner (package, version 0.6.2, and mapped reads were realigned around potential insertion/deletion (indel) sites with the Genome Analysis Tool Kit (GATK) version 1.6. Duplicate reads were marked using Picard version 1.107 and additional BAM file manipulations were performed with Samtools 0.1.18. Base quality (Phred scale) scores were recalibrated using GATK's covariance recalibration. SNP and indel variants were called using the GATK Unified Genotyper for each sample. All variants with potentially serious functional consequences (defined as nonsense, missense, or indel mutations within coding sequence, or those affecting essential splice sites) or affecting genes previously curated as part of the ciliary proteome (van Dam et al. 2013) were then selected.

For Sanger confirmation of mutations detected by exome sequencing, PCR amplification of genomic DNA was performed using M13‐tagged primers specific to the *BBS2* (NM_031885.3) gene. PCR products were examined by 1% agarose gel followed by Exo1/SAP treatment. Sequencing reactions were performed with M13 primers using BigDye terminator (4336919, Applied Biosystems) according to the manufacturer's protocol. Sequencing extension products were purified using BigDye cleaning beads (BCB‐100, MCLAB) and then analyzed with an ABI3730 DNA analyzer. DNA sequence data were analyzed with Sequencher software (Gene Codes Corporation, Ann Arbor, USA). Assessment of possible functional consequences of missense variants in ALMS1 was performed using the Combined Annotation Dependent Depletion (CADD) Tool v1.3 (http://cadd.gs.washington.edu/score) (Kircher et al. 2014).

### Statistical analysis

Statistical analyses were performed in GraphPad Prism 5.0 (GraphPad Software, San Diego, CA, USA). Statistical significance was determined by pairwise comparisons using a two‐tailed unpaired Student's *t* test with a *P* < 0.05 being considered significant. All data are presented as means ± SEM.

## Results

### Cohort studied

All volunteers studied had a clinical diagnosis of Alström syndrome, and were recruited from a dedicated multidisciplinary national clinical service (www.alstrom.co.uk) (Van Groenendael et al. 2015). A summary of the demographic characteristics of the cohort studied is given in Table S1. All had previously undergone diagnostic genetic testing of the *ALMS1* gene in different diagnostic laboratories, with 13 different nonsense mutations, 8 different frameshift mutations, and 5 different missense variants reported clinically (Table 1). Seventeen patients had biallelic nonsense or frameshift mutations, and two patients had heterozygous nonsense or frameshift mutations. Two patients were compound heterozygous for a frameshift or nonsense mutation and the same missense variant, p.Asn1787Asp, and one for a frameshift variant and the p.Asn2945Lys missense variant. Finally, one patient was compound heterozygous for two missense variants (p.His3881Tyr and p.Val423Ile) (Joy et al. 2007), and one patient was heterozygous for one missense variant (p.His624Arg). All variants had been reported as pathogenic on clinical testing.

**Table 1:** ALMS1 mutations, protein expression of ALMS1 in dermal fibroblasts, and clinical phenotypes of patients studied. All sequence variants are numbered according to canonical ALMS1 transcript ENST00000613296.4. Underlined mutations are predicted to truncate the ALMS1 protein before the epitope recognized by the anti‐ALMS1 antibody used for immunofluorescence. Missense mutations are shown in bold. Allele frequency of those mutations in the ExAC database is indicated in brackets after these

Patient	ALMS1 genotype	ALMS1 detection	Ciliogenesis	Vision	Hearing	Heart	Obesity?	Metabolism	Liver	Kidneys	Other
P1	p.Arg578Glyfs*17/p.Gln3494*	− PF ± M	Normal	NP 6 m RB 10y	BHA (15y)	Severe fibrosis IHD (35y)	Yes (childhood)	AN DM (14 y) Severe MDL	Fibrosis (25y)	CKD3 (30y)	Died pneumonia (39y)
P2	**p.Asn2945Lys** (1.0%)/p.Lys2196Serfs*10	± PF + M	Normal	NP 3 m VA 6/36 (24y)	BHA (10y)	Mild fibrosis	No (BMI 26, WHR 0.84 (23y))	AN (8y) DM (10y)^b^ Mild MDL	Fibrosis (23y)	Normal (23y)	
P3	p.Thr3591Lysfs*6/p.Arg3805*	− PF − M	Normal	NP 3 m RB 4y	BHA (10y)	PTCA (37y)	Yes (BMI 34, WHR 1.05 (38y))	AN (5y) DM (15y) Severe MDL	Fibrosis (34y)	CKD5 (38y)	Died pneumonia (43y)
P4	p.Arg2669*/p.Arg3628*	− PF − M	Normal	NP 6 m RB 10y	BHA (9y)	Severe fibrosis	No (BMI 28, WHR 0.92 (27y))	AN (6y) DM (14y) Mild MDL	Fibrosis (20y)	Normal	Kyphoscoliosis
P5	pGln3494*/p.Thr3591Lysfs*6	− PF − M	Normal	NP 48 m RB 15y	14y	Mild fibrosis	Yes (16y) (WHR 1.0 (45y))	AN DM (36y)	Fibrosis	CKD3 (42y)	Forestier's disease
P6	**p.His3881Tyr** (0.1%)/**p.Val423Ile** (0.3%)	± PF + M	Normal	NP 144 m RB 30y	Mild deafness No HA	Fibrosis (37y)	Yes (BMI 35, WHR 1.0 (33y))	AN DM (teens) MDL Severe IR	Fibrosis (36y)	RT (c.20y)	
P7	p.Ser3960Phefs*12/WT	± PF + M	Normal	NP 120 m RB 37y	Sensorineural deafness (18y) BHA 30y	Fibrosis (50y)	Yes (BMI 32.2, WHR 1.0 (25y))	AN severe IR Mild MDL	NAFLD Fibrosis	CKD3	2 brothers with cardiomyopathy, blindness, deafness
P8	p.Gln3000*/WT	− PF − M	Normal	NP 18 m RB 6y	7y	Normal	Yes (BMI 42, WHR 0.87 (40y))	Severe IR DM (35y)	NAFLD Fibrosis	CKD3 (42y)	
P9	p.Gln3816*/p.Val2300Trpfs*43	− PF − M	Normal	NP 12 m RB 5y	12y	Normal	Yes (BMI 34.4, WHR 1.0 (31y))	AN DM (18y)	NAFLD	No	Mild kyphoscoliosis.
P10	**p.His624Arg** (1.9%)/WT	+ PF + M	Normal	NP 360 m perceives light (43y)	Mild deafness (42y)	No	Yes	DM (30y)	Normal	No	Sibling died in infancy, heart failure.
P11	p.Thr3591Lysfs*6/**p.Asn1787Asp** (1.4%)	− PF − M	Normal	NP <2 m RB 10y	BHA	No	Yes	MDL IGT	NAFLD	No	Hypothyroid
P12	p.Ser1382*/p.Gln3000*	− PF − M	Normal	NP <3 m RB 10y	BHA	No	Yes	MDL IGT	NAFLD	No	
P13	p.Thr3591Ilefs*5/p.Thr3591Ilefs*5	− PF − M	Normal	NP <3 m RB 10y	BHA	Yes	Yes	No	No	No	
P14^a^	p.Ser1645*/p.Ser1645*	− PF − M	Normal	NP 1 m	BHA	No	Yes	No	NAFLD	No	
P15^a^	p.Ser1645*/p.Ser1645*	− PF − M	Normal	NP <3 m RB 13y	BHA	No	Yes	DM Severe MDL	Yes	No	
P16	p.Gln2979*/**p.Asn1787Asp** (1.4%)	− PF ± M	Normal	NP <3 m RB 12y	BHA	No	Yes	No	No	No	
P17	p.Leu940*/p.Thr2457Thrfs*18	− PF − M	Normal	NP <3 m	BHA	No	Yes	Yes	No	No	Kyphoscoliosis
P18	p.Thr3591 fs*6/p.Ser1948 fs	− PF − M	Normal	NP <3 m RB 7y	BHA	No	Yes	No	No	No	
P19	p.Thr3591 fs*6/p.Ser1948 fs	− PF − M	Normal	NP <3 m	BHA	Yes	Yes	No	No	No	
P20	p.Thr3591Lys fs*6/p.Gln3494*	− PF − M	Normal	NP <3 m	BHA	Yes	Yes	No	No	No	
P21	p.Trp266*/p.Arg3702*	± PF + M	Normal	NP 18 m	No	Yes (3 m) HT	No	No	No	No	Hypertension
P22	p.Arg2927*/p.Arg2927*	− PF − M	Normal	NP <3 m RB 11y	BHA	No	Yes	Yes	No	No	
P23	p.Ser1645*/p.Ser1645*	− PF − M	Normal	NP <3 m	No	Yes (CM)	Yes	No	No	No	

PF, paraformaldehyde fixation; M, methanol fixation. “+” = normal intensity of ALMS1 immunostaining; “−” = absent staining; “±” = weak staining. NP = age at development of nystagmus and/or photophobia. RB = age when registered blind; (B)HA = (Bilateral) hearing aids. Cardiac fibrosis was detected by MRI. HT, heart transplantation; BMI, body mass index; WHR, waist:hip ratio; AN, acanthosis nigricans; MDL, metabolic dyslipidemia, denoting elevated plasma triglyceride and suppressed HDL cholesterol; DM, diabetes mellitus; IR, insulin resistance; IGT, impaired glucose tolerance; NAFLD, nonalcoholic fatty liver disease; CKD, chronic kidney disease; RT, renal transplantation; PCR studies of ALMS1 cDNA showed no evidence of exon skipping for P6, P7, P10, P11, nor P15. P6 and P7 underwent exome sequencing as described in text, and compound heterozygous pathogenic BBS2 mutations were later found in P6.

a P14 and P15 are siblings.

b P2 became absolutely insulin deficient with detectable anti‐glutamic acid decarboxylase antibodies at 20 years old.

### ALMS1 expression and ciliogenesis in primary dermal fibroblasts

**Figure 1:**
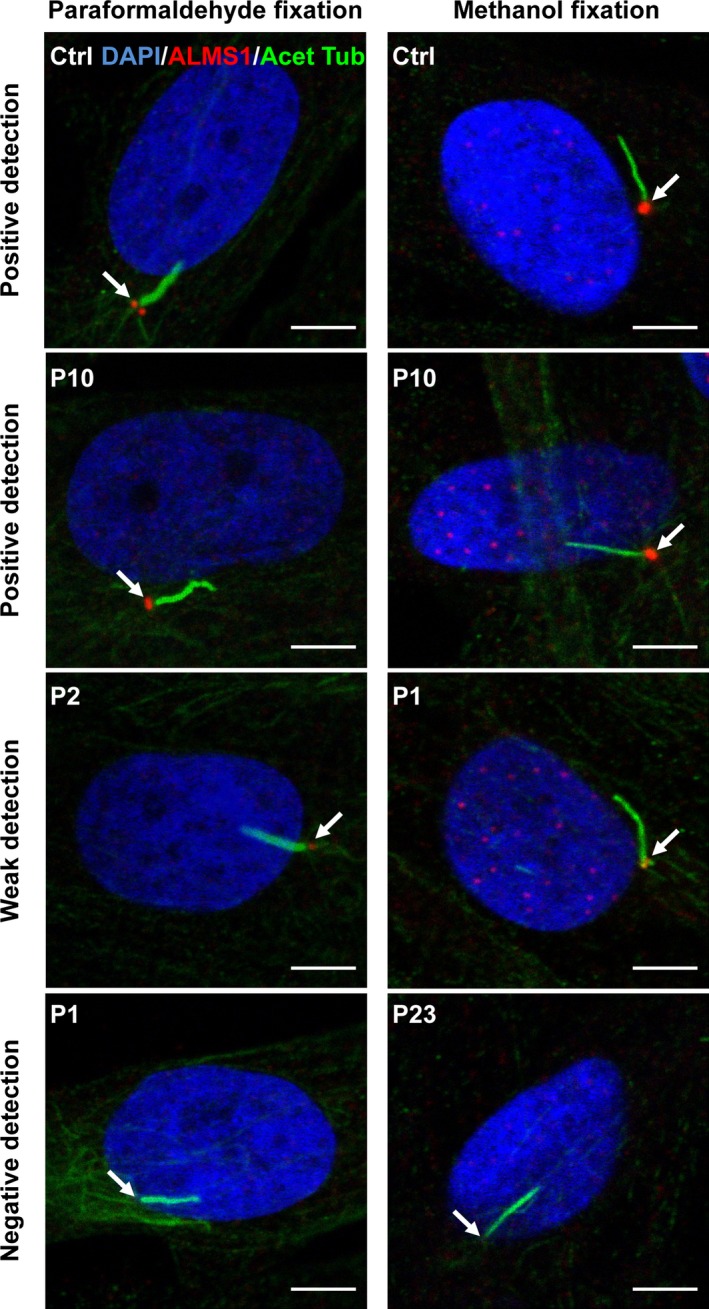
Representative immunofluorescent images of primary dermal fibroblasts (A) Cells fixed with 4% paraformaldehyde or methanol followed by coimmunostaining with anti‐ALMS1 and anti‐acetylated tubulin (Acet Tub). Arrows indicate localization of ALM1 which colocalizes with centrosomes/basal body of the primary cilium. Representative examples of appearances of “positive” (healthy control and P10), “negative” (P1 and P23), and “weak” (P2 and P1) detection are shown. Only merged images are shown. Individual channel and merged images as well as images stained with anti‐*γ*‐tubulin antibodies can be seen in Figure S1. Scale bars indicate 5 μm.

Immunofluorescent staining using two different cell fixation methods (namely 4% paraformaldehyde or methanol) and a specific anti‐ALMS1 antibody was first employed to examine ALMS1 expression in primary dermal fibroblasts derived from all volunteers studied. The ALMS1 antibody used has been employed in many previously studies, and was validated by showing loss of staining in HEK‐293 cells after shRNA‐mediated ALMS1 knockdown (data not shown). As the large majority of the ALMS1 mutations reported in this group produced premature stop codons resulting in truncated proteins, we selected an antibody that was raised against a synthetic peptide corresponding to a region within amino acids 1200–1250 of human ALMS1 to ensure that any surviving truncated proteins in cells with premature stop mutations after this point in the protein were detected. Figure 1 shows representative images for positive, negative, or weak positive ALMS1 detections in both paraformaldehyde‐ and methanol‐fixed cells. We noticed that ALMS1 protein expression can be more readily detected in methanol‐fixed cells compared to paraformaldehyde‐fixed cells. For example, in methanol‐fixed P1 and P16 cells, ALMS1 was readily visible (although weak compared to positive controls) but in the paraformaldehyde‐fixed cells, ALMS1 showed a negative detection (see Figure S2B,C for ALMS1 staining intensities measured in representative positive, negative, and weak ALMS1‐expressing cells). Only three of the convincing truncating mutations in the patients studied occurred before this epitope (Table 1). Of 23 cell lines assessed, 16 (69.6%) showed no evidence of ALMS1 staining after either paraformaldehyde or methanol fixation; 14 of these cell lines harbored biallelic nonsense or frameshift mutations, while the remaining line from patient 8 (P8) harbored only one convincing pathogenic mutation. Two out of twenty‐three (8.7%) lines, one harboring a frameshift and a nonsense mutation (P1), and one with a nonsense mutation and a missense variant (P16; the missense variant is p.Asn1787Asp; found in 1.4% of alleles in the ExAC exome dataset) showed equivocal staining for ALMS1 protein. Notably, however, cells from P11, harboring the same p.Asn1787Asp variant together with a frameshift mutation (p.Thr3591Lysfs*6) showed no immunodetectable ALMS1 expression; 5/23 (21.7%) lines showed normal ALMS1 expression using one of two fixation methods. One of these (P21) had biallelic nonsense or frameshift mutations, one (P2) was compound heterozygous for a frameshift mutation and a missense variant (p.Asn2945Lys), one was heterozygous for a single frameshift mutation (P7), and two had only three missense variants between them (P6 was compound heterozygous for p.His3881Tyr and p.Val423Ile (Joy et al. 2007), and P10 was heterozygous for p.His624Arg). The missense variants had been deemed to be pathogenic in clinical testing, but were found at allele frequencies of between 0.1 and 1.9% in the ExAC exome dataset (Table 1) (Lek et al. 2016).

Immunocytochemistry thus stratified the patients studied into three groups defined by absent ALMS1 detection, equivocal ALMS1 detection (about 30% of normal ALMS1 staining intensity, e.g., in methanol‐fixed P1 and P16 cells, see Figure S2B), and normal ALMS1 detection using one of two fixation protocols. In order to see whether the variability between the expression levels of nonsense mutations in ALMS1 (e.g., in P1, P16) is related to differences in the extent of nonsense‐mediated mRNA decay (NMD) (Lindeboom et al. 2016) of the ALMS1 mRNA, we performed TaqMan assay using two sets of primers/probes, spanning, respectively, exon 4 and exon 5, and exon 13 and exon 14 of ALMS1 gene (see Figure S3). Indeed, the relative expression levels of ALMS1 mRNA in P6, P10, and P21 which all showed positive ALMS1 protein detections in immunocytochemistry were higher than the rest of the lines analyzed. The relative expression levels of ALMS1 mRNA in P5, P8, P18, P22, and P23 (all showed negative ALMS1 protein detections) were relatively low (Figure S3). It is therefore conceivable that the variability between the expression levels of nonsense mutations in ALMS1 might be related to differences in the extent of nonsense‐mediated mRNA decay (NMD) of the ALMS1 mRNA (Lindeboom et al. 2016). Correlations between ALMS1 genotype and cell phenotype on one hand, and between clinical phenotype and cell phenotype on the other, were then assessed. Although the age at onset of many components of the syndrome was not precisely documented in most cases, age at onset of nystagmus, photophobia, and deafness were accurately recorded in clinical files. The age at the former was substantially later in patients with normal ALMS1 staining (median 120 (range 3–360) months compared to <3 (1–48) in those with no detectable ALMS1 protein), and hearing was also relatively preserved compared to those with no ALMS1 staining. Moreover even in P2, whose cells showed normal ALMS1 staining, and who was recorded to have nystagmus and photophobia around 3 months old, visual acuity remained at 6/36 at the age of 24 years old, highly atypically for Alström syndrome. No clear differences in other components of the syndrome were discernible, however.

Viewed from the perspective of molecular genetic testing results, 14 of the 16 cell lines with biallelic nonsense or frameshift mutations in ALMS1 had undetectable ALMS1 staining, either focally at the centrosome or more diffusely in the cell, suggesting that a large majority of truncating ALMS1 alleles are not expressed at the protein level. In one line, nearly normal staining was seen, surprisingly, while in one further line equivocal staining was observed. In both cases, this staining was seen at the centrosome rather than diffusely distributed. The two patients with clearly visible ALMS1 cellular staining did have a later onset or more indolent course of visual symptoms.

Two cell lines harbored a heterozygous nonsense or frameshift mutation, with no second mutation reported in either. ALMS1 expression was undetectable in the cells with the heterozygous nonsense mutation, from P8, consistent with the presence of an undetected second mutation abolishing expression. The cells which had a heterozygous frameshift mutation, from P7, showed nearly normal ALMS1 staining, in contrast. This patient had a milder phenotype than other patients with convincing biallelic ALMS1 mutations, and also reported two siblings with cardiomyopathy, blindness and deafness. No family members were available for study.

Five cell lines had been reported to harbor at least one pathogenic missense mutation. Two, P11 and P16, had the same missense variant, p.Asn1787Asp, coinherited with either a frameshift or a nonsense mutation, while P2 has the p.Asn2945Lys variant coinherited with a frameshift mutation. In P11, no ALMS1 staining was seen, in P16, staining was only equivocal, and in P2, it was nearly normal; however, the associated clinical syndromes were each typical of Alström syndrome. Finally, two cell lines were from patients in whom only missense variants in ALMS1 had been reported. One line was established from P6, a previously published patient with compound heterozygous ALMS1 variants, p.His3881Tyr and p.Val423Ile, while the second was from P10, a patient who was heterozygous for the p.His624Arg variant only. All three of these variants have been shown to be relatively common in large populations (0.1%, 0.3% and 1.9%, respectively, in ExAC), a panel of predictive algorithms predicted none of them to be pathogenic (Table S3), and ALMS1 expression in both cell lines harboring these variants was normal. The age of onset of eye symptoms of Alström syndrome was strikingly later in these patients than the rest of the cohort.

As well as using anti‐ALMS1 antibody to stain ALMS1 in all 23 cell lines studied, an anti‐acetylated tubulin antibody was also used to stain cytoplasmic microtubules and ciliary axonemes, with anti‐γ‐tubulin antibody to stain centrosomes (Hearn et al. 2005). All cell lines showed normal primary cilia formation with no discernible defects in ciliary morphology compared to healthy control cells (Fig. 1 and Table 1; also see Figure S2A for ciliary length measured in selected representative AS cell lines).

### Exome sequencing in atypical patients

Based on preserved ALMS1 detection in cellular studies, mild and/or atypical clinical phenotypes, and lack of two convincingly pathogenic ALMS1 mutations, it was hypothesized that volunteers P6, P7, and P10 may harbor additional, or alternative, pathogenic mutations that had not been detected hitherto. Two of these three volunteers, patients P6 and P7, consented to exome‐wide sequencing to address this possibility.

**Figure 2:**
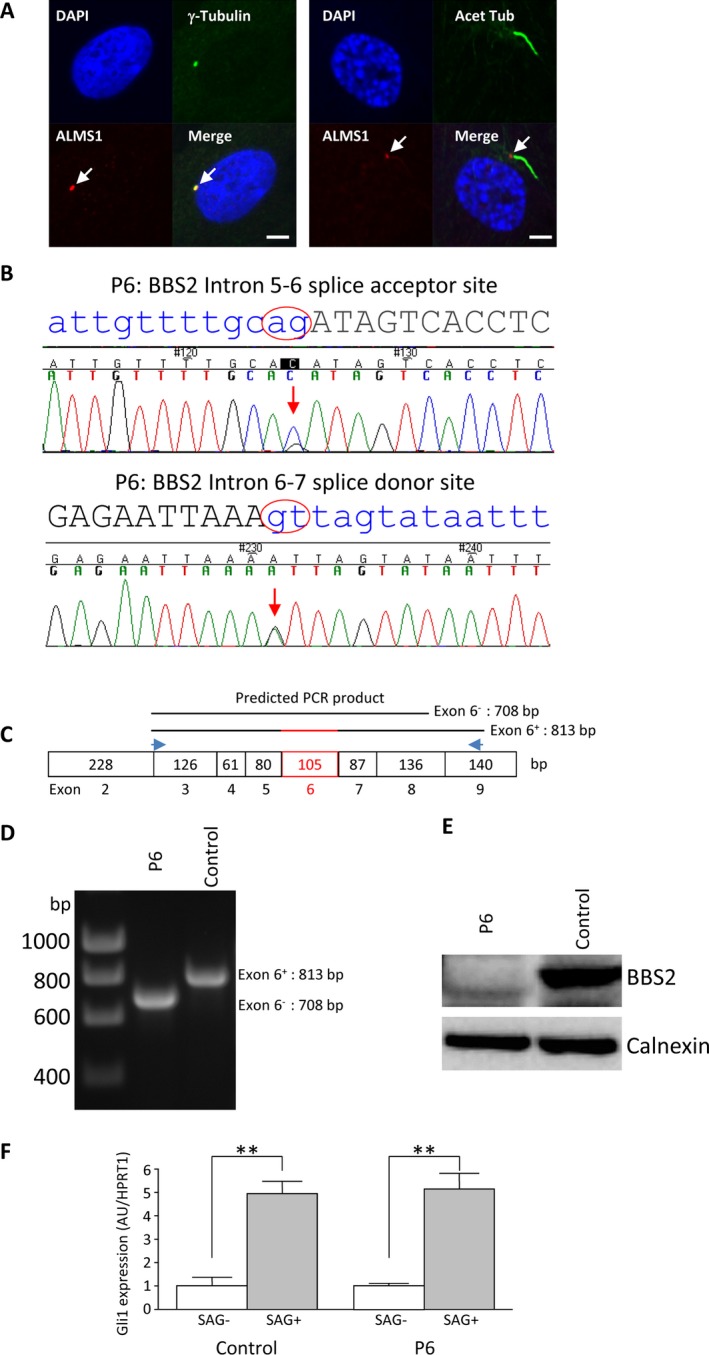
Analysis of P6 dermal fibroblasts. (A) Immunofluorescence staining of P6 fibroblasts revealing positive detection of ALMS1 and the primary cilium. Scale bars indicate 5 μm. (B) Sanger sequencing confirmation showing heterozygous mutations at the *BBS2* (NM_031885.3) intron 5–6 splice acceptor site and intron 6–7 donor site. (C) Schematic representation of PCR analysis strategy, with a pair of primers flanking exon 6 giving rise to a product of predicted size 813 base pairs (bp) for the wild type and 708 bp for the mutant gene. (D) 1% agarose gel revealed a single PCR product of predicted size for control and the patient cells, respectively. (E) Lack of wild type BBS2 protein in the patient cells, as opposed to the control cells revealed by western blot analysis. (F) qPCR analysis of *GLI1* expression (arbitrary unit: AU) in control and patient cells following 24 h serum starvation and subsequent 24 h SAG treatment with housekeeping gene *HPRT1* as loading control. Results are reported as mean values ± SEM and are expressed as fold change with respect to untreated controls, arbitrarily set as 1; **P < 0.01.

In patient P6, whose clinical syndrome had previously been reported and attributed to the ALMS1 missense mutations (Joy et al. 2007), this analysis revealed two rare and highly likely pathogenic heterozygous mutations in *BBS2*, one affecting the intron 5 splice acceptor site (c.613‐1G>C) and the other the intron 6 splice donor site (c.717 + 1G>A). The mutations were confirmed by Sanger sequencing of genomic DNA (Fig. 2B). PCR amplification of cDNA derived from fibroblasts using primers flanking exon 6 of *BBS2* revealed that exon 6 was absent (Fig. 2C,D), indicating that the mutations identified were on different alleles, and also abolishing normal exon 6 splicing, while western blotting showed no expression of full length BBS2 protein in the cells (Fig. 2E). A weakly detected smaller band that was not present in control cells may, however, represent low levels of expression of mutant BBS2 protein lacking exon 6 (Fig. 2E).

In patient P7, in contrast, no convincing biallelic mutations were identified in any known human ciliopathy gene, although the previously detected ALMS1 p.Ser3960Phefs*12 mutation was confirmed together with a common single amino acid insertion in a minor transcript of ALMS1 (rs147096460; p.524insPro; allele frequency 0.63 in ExAC). To address the possibility of a digenic cause of the patient's phenotype, exome sequence data were interrogated to identify all rare variants in genes assembled in a highly curated ciliary proteome (van Dam et al. 2013). Eleven homozygous variants were thus identified, while in three genes aside from ALMS1, two or more different variants were detected. Possible compound heterozygous and a subset of the most damaging of the other mutations were confirmed by Sanger sequencing. However, while the genetic background of possible contributing mutations in ciliary genes was thus defined, as shown in Table S2, no convincing digenic effect could be established by study of one patient alone. No copy number variants were identified in any ciliary gene on analysis of exome data.

### Hedgehog signaling in dermal fibroblasts

In mammalian cells, Hedgehog signaling requires the primary cilium. The Hedgehog receptor Patched 1 (PTCH1) is enriched in and around the primary cilium, where it inhibits Smoothened (SMO) activity. In the absence of sonic hedgehog (SHH), at the base of cilia, the GLI1 proteins GLI2 and GLI3 are phosphorylated by a kinase complex consisting of PKA, CSNK1A1 (aka CK1), and GSK3B. This results in their proteolytic cleavage to generate the repressor forms (GLI2R and GLI3R, respectively). In the presence of SHH ligand, PTCH1 is internalized and degraded; as a result SMO is phosphorylated and translocated into the primary cilium where it helps preventing the GLI proteins from proteolytic processing. The full length, activated GLI proteins (GLI2A and GLI3A, respectively) eventually migrate into the nucleus and activate target gene expression (Briscoe and Therond 2013). Transcription of *GLI1* in response to hedgehog is thus a widely used biomarker for increased canonical hedgehog signaling activity (Robbins et al. 2012).

**Figure 3:**
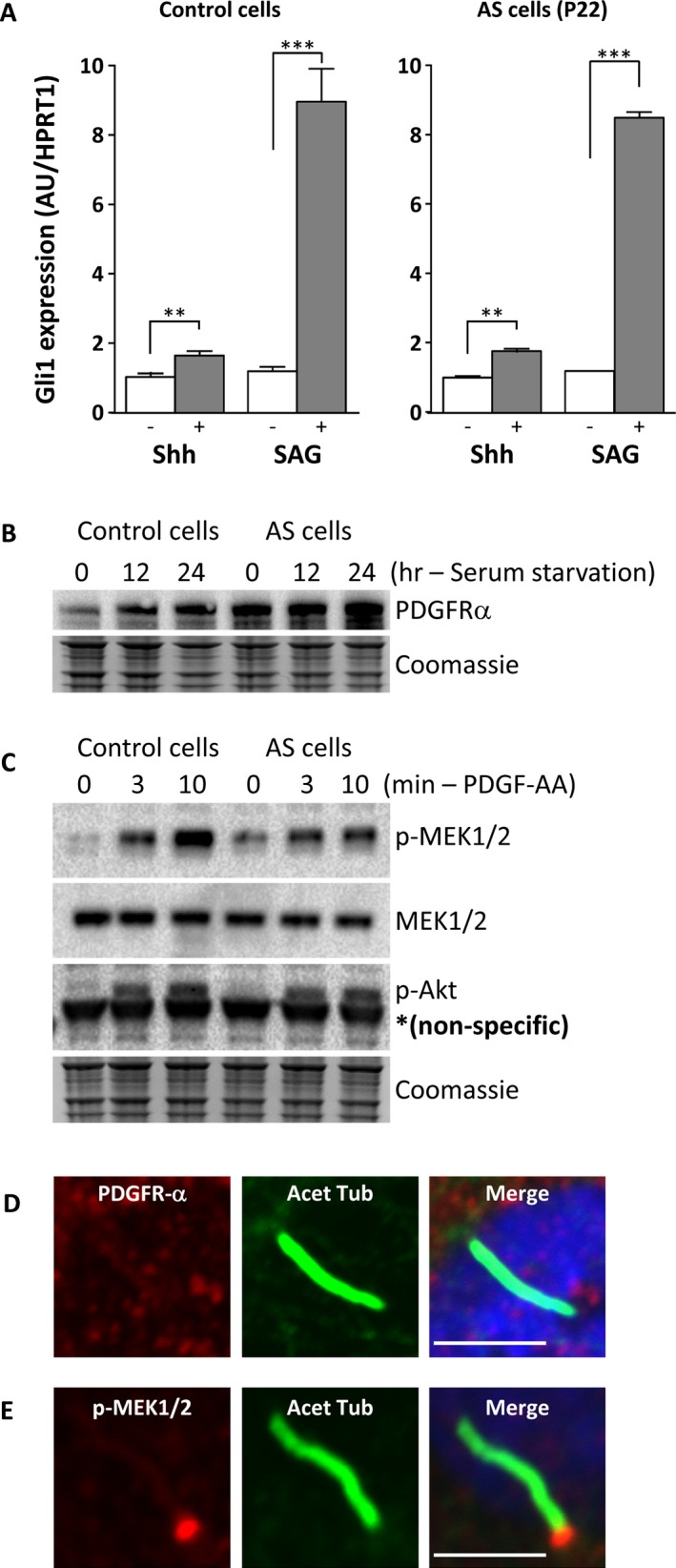
Hedgehog and PDGFA signaling assay (A) Transcriptional induction of GLI1 in serum‐starved cells exposed to Hedgehog (SHH) or Smoothened agonist SAG treatment for 24 h. Dermal fibroblasts derived from one control and one patient (P22) which showed no ALMS1 detection by immunofluorescent staining were used. *GLI1* expression was determined by quantitative RT‐PCR and is expressed in arbitrary units (AU). **P < 0.01, ***P < 0.001. (B) Immunoblotting for PDGFRA of cells harvested at the indicated times during serum starvation. (C) Phosphorylation of MEK1/2 and AKT in response to PDGF‐AA treatment of serum‐starved cells. Loading was assessed by Coomassie blue staining. (D) Localization of PDGFRA and phospho‐MEK1/2 in serum‐starved dermal fibroblasts assessed by immunofluorescent staining (E) Localization of PDGFRA and phospho‐MEK1/2 in serum‐starved dermal fibroblasts treated with PDGF‐AA. Scale bars indicate 5 μm.

To assess whether Hedgehog signaling is compromised by lack of ALMS1 at the basal body of cilia in dermal fibroblasts, we assessed the response to serum starvation followed by SHH or the SHH pathway agonist SAG treatment of healthy control cell line, an AS cell line with no detectable ALMS1 expression, and cells from the patient we identified with biallelic BBS2 mutations. We analyzed SMO translocation, proteolytic cleavage of GLI proteins, and GLI1 expression by, respectively, western blotting and qRT‐PCR. No proteolytic processing of GLI proteins was detected in any of the cells studied, likely due to low GLI protein expression. However, GLI1 mRNA expression was increased significantly in all three cell lines by SHH (twofold increase) or SAG treatment (ninefold increase) (Figs 3A and 2F). These findings not only suggest that ALMS1 protein at the basal body of the primary cilium is not critically involved in Hedgehog signaling, but also that the BBS2 mutations we describe in patient P6 are not sufficient to abolish ciliary function (Fig. 2F).

### PDGFA signaling in dermal fibroblasts

As a further test of cell signaling mediated by the primary cilium, we also examined PDGFA signaling in control and ALMS1‐deficient cells, as PDGFA signaling has been shown to be regulated through the primary cilium in fibroblasts (Schneider et al. 2005). Healthy control and AS patient dermal fibroblasts were serum starved for 24 h followed by treatment with PDGF‐AA. As shown in Fig. 3B, PDGFRA (platelet‐derived growth factor receptor alpha) was upregulated by serum starvation in both healthy control and patient cells. Localization of PDGFRA to primary cilia was not evident, however (Fig. 3D). Phosphorylation of downstream signaling molecules such as MEK1/2 and AKT upon stimulation with PDGF‐AA was observed in both control and AS patient cells (Fig. 3C). Moreover, similar to prior observations (Schneider et al. 2005) phosphorylated MEK1/2 was detected mainly at the basal body with some in the primary cilia as well (Fig. 3E). No difference in phospho‐MEK1/2 localization was observed between healthy control and AS patient cells. Taken together, these findings suggest that ALMS1 is not critically involved in the PDGFA signaling pathway.

## Discussion

We have studied 23 patients attending a national specialized clinic for Alström syndrome (Van Groenendael et al. 2015). The spectrum of clinical severity was approximately in keeping with prior reports of the natural history of the syndrome (Michaud et al. 1996; Russell‐Eggitt et al. 1998; Marshall et al. 2005; Mokashi and Cummings 2011; Paisey 2013). Disease severity is difficult to quantify, however, with many of the pleiotropic features of the syndrome remaining occult until detailed medical assessment, meaning that time of onset is often imprecisely established. In most patients nystagmus and photodysphoria related to retinal disease are noticed early and remembered, and these serve as a potentially useful semiquantitative index of disease severity. We have now assessed this index of disease severity in the context of cellular studies as well as molecular genetic testing, allowing testing of putative genotype–phenotype correlations with a higher degree of confidence than has been possible hitherto in studies where the consequence of the nucleotide change for protein expression was uncertain.

Truncating mutations may give rise to proteins that are unstable, and thus abolish protein expression, or may give rise to stable truncated proteins with perturbed function. In the case of ALMS1, loss of the centrosome‐targeting domain at the C terminus would be expected to lead to preservation of cellular ALMS1 staining, but with a diffuse pattern, if the truncated protein were expressed (Knorz et al. 2010). In 14 of the 16 cell lines we studied with biallelic nonsense or frameshift ALMS1 mutations, however, no ALMS1 staining was visible, consistent with absent protein expression. In one line nearly normal staining was seen, surprisingly, while in one further line equivocal staining was observed. It was not possible to discern whether this was due to expression of a truncated protein, or readthrough (Dabrowski et al. 2015) of the premature stop codon to yield full length protein. The patient with clearly visible ALMS1 cellular staining, although having unequivocal Alström syndrome, did show a later onset and more indolent course of visual symptoms.

Many examples of patients with a clinical diagnosis of Alström syndrome but with only one convincing pathogenic mutation have been reported (e.g., Marshall et al. 2015), and it is usually assumed that another undetected mutation, most likely either affecting a noncoding *cis* regulatory element, or resulting in a larger intragenic indel, is present. We found undetectable ALMS1 expression in one cell line with a heterozygous nonsense mutation, consistent with this; however, the other cell line with a heterozygous frameshift mutation showed nearly normal ALMS1 staining. Exome‐wide sequencing in this patient showed only a common polymorphism in a minor ALMS1 splice variant, but did delineate a range of other sequence variants present in known ciliopathy genes. This patient had a milder phenotype than other patients with convincing biallelic ALMS1 mutations, and also reported two siblings who were not available for study with cardiomyopathy, blindness, and deafness. Although lack of an extended family to study precludes formal investigation of a digenic interaction, this remains a possible explanation for the syndrome in this family.

Assigning pathogenicity of missense mutations in Alström syndrome is very challenging, partly because of the very large number of rare missense alleles reported in large publically available datasets such as the ExAC dataset (Lek et al. 2016), and partly because no simple functional assay exists with which to assess the consequence of such variants. Two of the cases we describe, with typical Alström syndrome, had the same missense variant, p.Asn1787Asp, coinherited with either a frameshift or a nonsense mutation. In one case no ALMS1 staining was seen, while in the other staining was only equivocal. Thus, although this variant is relatively common, being identified at an allele frequency of 1.4% in the Exome Aggregation Consortium (ExAC) dataset (Lek et al. 2016), and although it is not predicted to be deleterious by a panel of predictive algorithms, we suggest that it may confer significant loss of function through destabilizing the ALMS1 protein. In a second cell line the p.Asn2945Lys variant, seen at 1% in ExAC, was found together with a frameshift mutation, and ALMS1 expression was near normal despite typical Alström syndrome with the exception of visual acuity that remained at 6/36 at the age of 24 years old. This would be consistent with the p.Asn2945Lys variant being expressed but dysfunctional, although it is predicted to be benign by a panel of algorithms. The formal possibility of linkage to an undetected *cis* acting mutation in all three of these cases, or interaction with a defect in a second gene, cannot be excluded, however.

In the two patients with a prior clinical diagnosis of Alström syndrome but only missense variants in ALMS1, ALMS1 expression in cells was normal. Furthermore, the age of onset of cardinal features of Alström syndrome was strikingly later than in the rest of the cohort. Collectively, these findings argue against pathogenicity of the ALMS1 missense mutations previously said to be pathogenic (Joy et al. 2007), and indeed in the one patient who agreed to further evaluation with exome‐wide sequencing, an alternative genetic diagnosis, Bardet–Biedl syndrome (BBS) due to convincing compound heterozygous splice site mutations in BBS2, was established. Such overlap between Alström and Bardet–Biedl syndrome has previously been reported to lead to a significant degree of clinical misclassification of the two disorders (Deveault et al. 2011; Redin et al. 2012).

Ciliogenesis requires a functional intraflagellar transport (IFT) system to shuttle building proteins for construction and maintenance of the cilium. Loss of IFT complex protein(s) often results in short or absent cilia (Ishikawa and Marshall 2011). Our observation that cilia in all Alström syndrome cell lines examined in this study appeared normal confirms prior findings (Collin et al. 2005; Hearn et al. 2005; Jagger et al. 2011) and demonstrates that ALMS1 is not required for ciliogenesis in dermal fibroblasts. While this could be taken to imply that ALMS1 is not critically involved in the IFT cargo complex as previously postulated (Girard and Petrovsky 2011), the role of ALMS1 in ciliary formation and/or function may be cell‐type‐ and/or tissue‐specific. Indeed knockdown of ALMS1 in either retinal pigment epithelial cells (Graser et al. 2007) or a mouse kidney epithelial cell line (Li et al. 2007) has been reported to produce stunted cilia without affecting the efficiency of ciliogenesis, and homozygous mutation of Alms1 in vivo in mice resulted in age‐dependent loss of cilia in the renal cortex (Li et al. 2007) and reduced the number of hypothalamic neuronal cilia (Heydet et al. 2013). It remains possible that in humans, too, these tissues exhibit abnormal ciliary structure.

The observation that Alström patient cells showed normal *GLI1* upregulation upon SHH and SAG stimulation further suggests that ALMS1 is not critically involved in the hedgehog signaling pathway in these cells. Similarly, unhindered phosphorylation of MEK1/2 and AKT in Alström patient cells upon PDGF‐AA stimulation suggests that ALMS1 is also not critically involved in PDGFA signaling pathway. This suggests that ALMS1 is dispensable for signal transduction in at least some well‐known cilia‐associated pathways. It has been noted, however, that ciliary localization is not a sufficient basis for assigning all functions of a protein to the cilium (Yuan and Sun 2013). Many ciliary proteins, including IFT components, are not exclusive to the cilium and have functions outside of the organelle (Yuan and Sun 2013). Indeed, a recent report demonstrated that ALMS1 might be involved in the endosome recycling by interacting with α‐actinin and components of the endosome recycling pathway (Collin et al. 2012). It is also interesting to note that primary dermal fibroblasts from patients with Alström syndrome do show abnormal phenotypes related to extracellular matrix formation and migration, although it remains unclear whether these relate to ciliary or nonciliary functions of the protein (Zulato et al. 2011).

In summary, our findings suggest that a large majority of loss‐of‐function mutations in ALMS1 result in a failure of protein expression, arguing that genotype–phenotype correlations previously suggested are unlikely to hold. Cells from occasional patients with biallelic loss of function ALMS1 mutations do exhibit preserved ALMS1 expression, however, and this corresponds to later onset or more indolent course of some key components of the syndrome. Our studies are consistent with the relatively common missense variant p.Asn1787Asp conferring loss or severe reduction in ALMS1 expression, suggest (but do not prove) that p.Asn2945Lys may be expressed but dysfunctional, but argue, conversely, that p.His624Arg, p.His3881Tyr, and p.Val423Ile are not pathogenic. We suggest that extreme caution should be exercised in assigning pathogenicity to *ALMS1* missense variants, and, given the overlap in clinical features among different ciliopathies, that wider analysis of a ciliopathy gene panel is warranted in any patient with features thought to denote Alström syndrome but without two convincing loss‐of‐function *ALMS1* mutations, especially where the phenotype is atypical or mild. In cases where this fails to yield a convincing genetic diagnosis, immunostaining for ALMS1 in primary cells may have utility.

## Conflict of Interest

The authors report no conflicts of interest.

## Supporting information


**Figure S1.** Representative immunofluorescent images of dermal fibroblasts.
**Figure S2.** Quantification of ciliary length and ALMS1 expression in representative primary dermal fibroblasts.
**Figure S3.** ALMS1 mRNA expression in selected primary dermal fibroblasts.
**Figure S4.** cDNA sequencing of dermal fibroblasts of P7, P8, P10, and P21.

**Table S1.** Demographic characteristics of patients studied.

**Table S2.** Rare variants in ciliary genes identified by exome sequencing of P7.

**Table S3.** A summary of scores obtained in assessing the missense variants in ALMS1 using the CADD (Combined Annotation Dependent Depletion) Tool v1.3.

**Appendix S1.** Supplementary Materials and Methods.
