# Supplementary material for: Refining genotype–phenotype correlation in Alström syndrome through study of primary human fibroblasts
Source: Mol Genet Genomic Med. 2017 May 15;5(4):390–404. doi: 10.1002/mgg3.296 (PMC5511801; doi:10.1002/mgg3.296)
Supplement: Supplementary file 1 — Figure S1. Representative immunofluorescent images of dermal fibroblasts. Figure S2. Quantification of ciliary length and ALMS1 expression in representative primary dermal fibroblasts. Figure S3. ALMS1 mRNA expression in selected primary dermal fibroblasts. Figure S4. cDNA sequencing of dermal fibroblasts of P7, P8, P10, and P21. [file MGG3-5-390-s001.docx]

**Supplementary Figures**

**
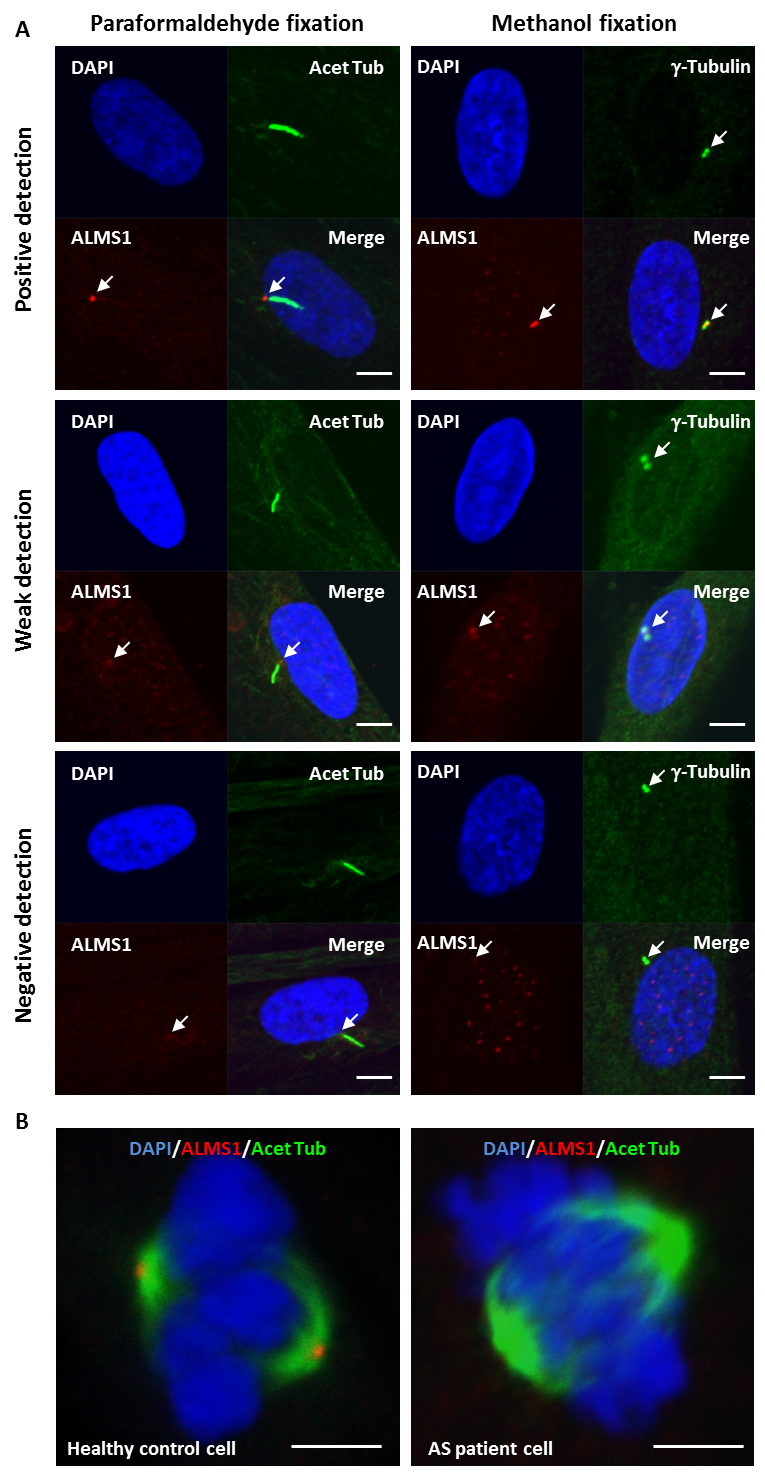
**

**Supplementary Figure S1. Representative immunofluorescent images of dermal fibroblasts.** (A) Cells fixed with 4% paraformaldehyde or methanol co-immunostained with anti-ALMS1 and either anti-acetylated tubulin (Acet Tub) or anti-γ-tubulin antibodies. Arrows indicate localization of ALMS1 to centrosomes/basal bodies of primary cilia. Representative “positive” (P10), “negative” (P23) and “weak” (P2) staining is shown. (B) Bipolar mitotic spindles in healthy control or AS patient (P23) cells showing absent ALMS1 in paraformaldehyde-fixed cells. Scale bar = 5 μm.

**
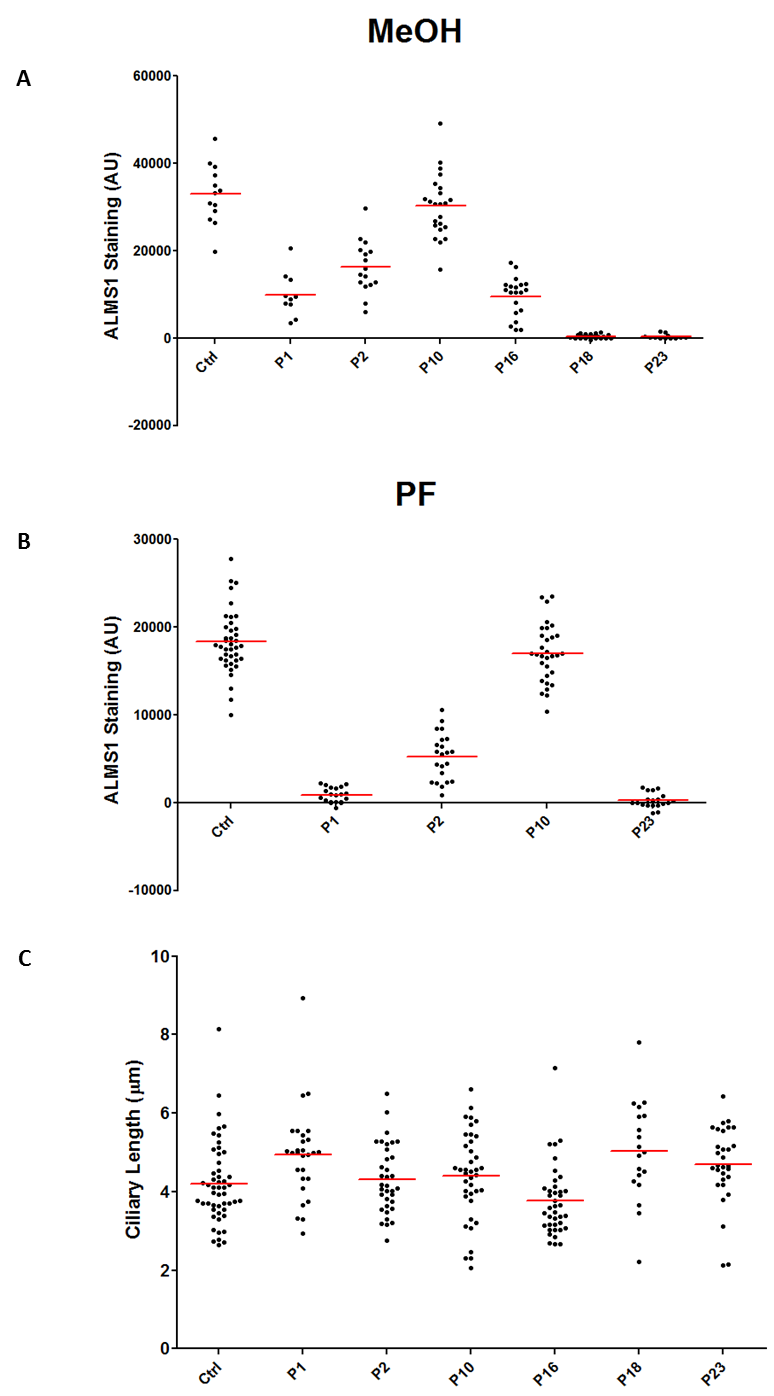
**

**Supplementary Figure S2. Quantification of ciliary length and ALMS1 expression in representative primary dermal fibroblasts.** Quantification of ALMS1 immunostaining in (A) methanol (MeOH)-fixed and (B) paraformaldehyde (PF)-fixed primary dermal fibroblasts. (C) Quantification of primary cilia length after immunostaining with anti-acetylated tubulin. Dots represent cells, and red lines indicate mean values.

**
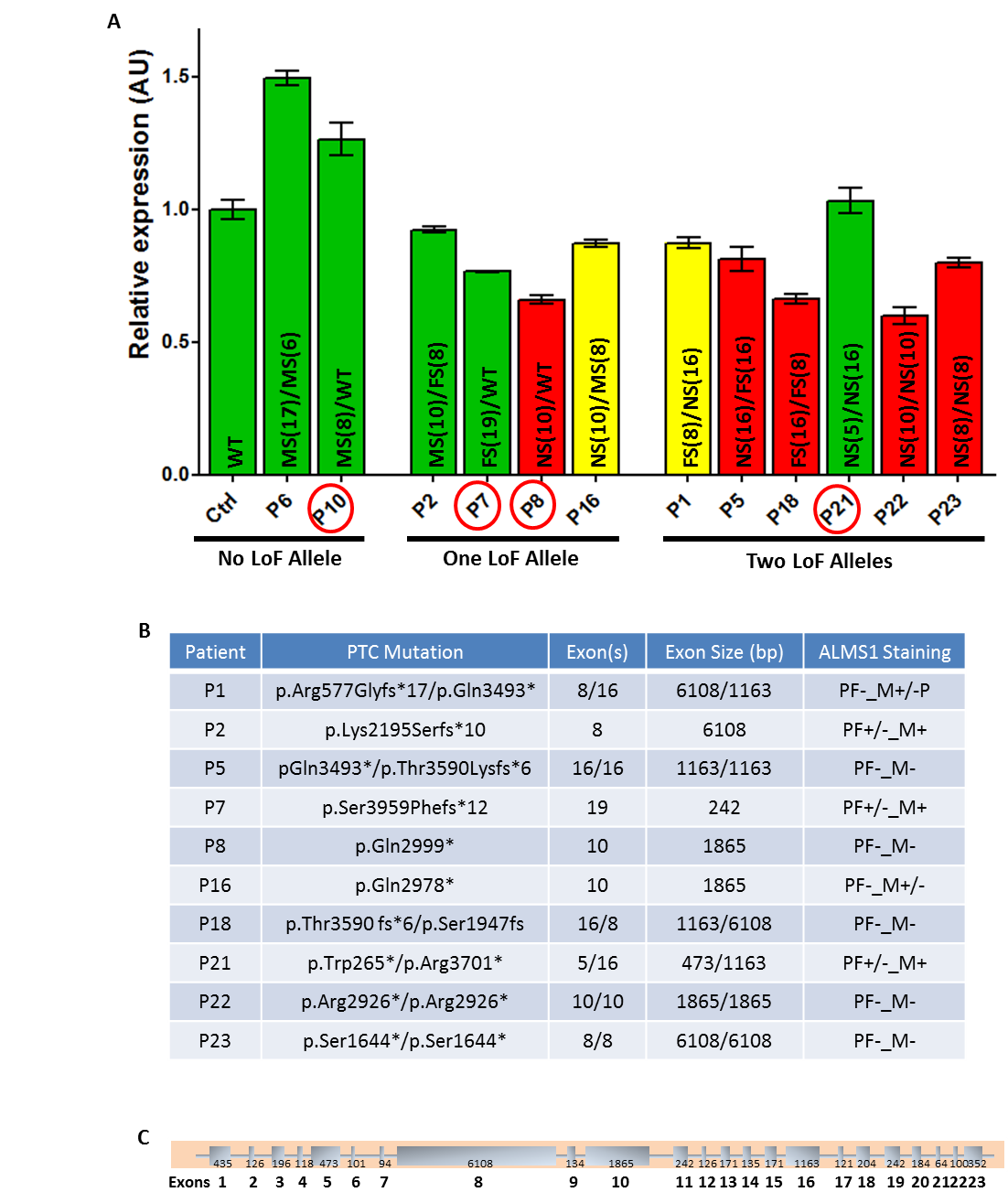
**

**Supplementary Figure S3. ALMS1 mRNA expression in selected primary dermal fibroblasts.** (A) ALMS1 expression normalised to HPRT1 in primary dermal fibroblasts determined by quantitative real time PCR. Error bars represent S.E.M of 3 technical replicates. Cells are grouped on the X axis by genotype according to number of nonsense or frameshift (“LoF”) alleles in ALMS1 (MS, missense; FS, frameshift; NS, Nonsense). Green shading denotes positive detection of ALMS1 by immunocytochemistry, yellow weak detection and red absent detection. Cells circled in red were also subjected to cDNA sequencing at mutation sites, as shown in Supplementary Figure S4. (B) Exon location of premature termination codons (PTC) in patients as well as ALMS1 immunostaining results. PF: paraformaldehyde fixation; M: methanol fixation; + denotes positive detection, - negative detection and +/- weak positive detection.


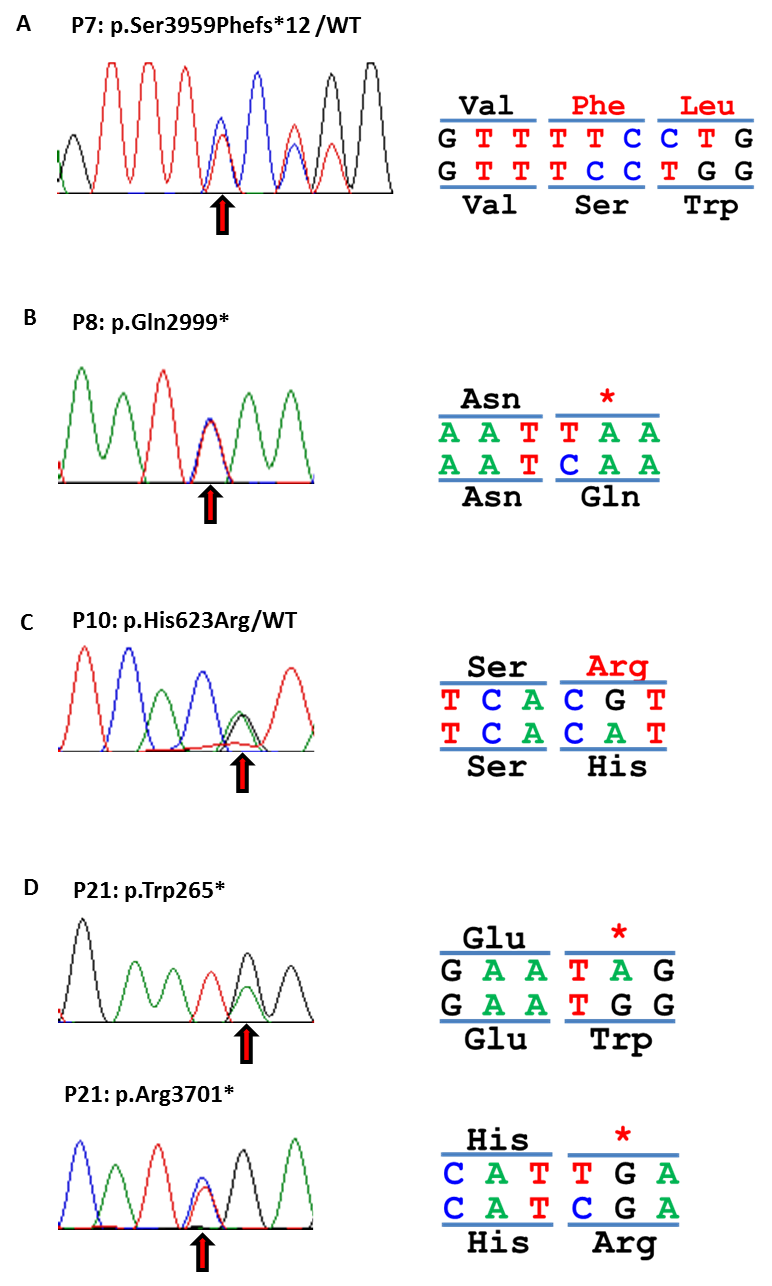


**Supplementary Figure S4. cDNA sequencing of dermal fibroblasts of P7, P8, P10 and P21.** Chromatograms of mutation sites are shown on the left hand side and the corresponding sequences represented on the right hand side with mutant allele and translation on top, and wild type allele with translation on the bottom.
