# Supplementary material for: Refining genotype–phenotype correlation in Alström syndrome through study of primary human fibroblasts
Source: Mol Genet Genomic Med. 2017 May 15;5(4):390–404. doi: 10.1002/mgg3.296 (PMC5511801; doi:10.1002/mgg3.296)
Supplement: Supplementary file 2 — Table S1. Demographic characteristics of patients studied. [file MGG3-5-390-s002.doc]

**Supplementary Tables**

| **Identifier** | **Sex** | **Age at time of study, years** | **Ethnicity** |
| --- | --- | --- | --- |
| P1 | Male | 38 | White British |
| P2 | Female | 20 | White British |
| P3 | Male | 42 | White British |
| P4 | Female | 27 | White British |
| P5 | Male | 48 | White British |
| P6 | Female | 37 | White British |
| P7 | Male | 59 | White British |
| P8 | Male | 41 | White British |
| P9 | Male | 27 | White British |
| P10 | Male | 44 | White British |
| P11 | Male | 15 | White British |
| P12 | Female | 9 | White British |
| P13 | Male | 10 | White British |
| P14 | Female | 7 | South Asian UK |
| P15 | Male | 18 | South Asian UK |
| P16 | Male | 17 | White British |
| P17 | Male | 13 | White Irish |
| P18 | Female | 5 | White British |
| P19 | Female | 5 | White British |
| P20 | Female | 4 | White British |
| P21 | Female | 7 | White British |
| P22 | Male | 11 | South Asian UK |
| P23 | Male | 7 | South Asian UK |

**Supplementary Table S1: Demographic characteristics of patients studied.**
