# Supplementary material for: Refining genotype–phenotype correlation in Alström syndrome through study of primary human fibroblasts
Source: Mol Genet Genomic Med. 2017 May 15;5(4):390–404. doi: 10.1002/mgg3.296 (PMC5511801; doi:10.1002/mgg3.296)
Supplement: Supplementary file 4 — Table S3. A summary of scores obtained in assessing the missense variants in ALMS1 using the CADD (Combined Annotation Dependent Depletion) Tool v1.3. [file MGG3-5-390-s004.docx]

| **rsID** | **Variant** | **Grantham** | **PolyPhen Category** | **PolyPhen Value** | **SIFT  category** | **SIFT value** | **Raw CADD  Score** | **PHRED-like  Scaled CADD Score** |
| --- | --- | --- | --- | --- | --- | --- | --- | --- |
| rs45630557 | V423I | 29 | benign | 0.009 | tolerated | 0.53 | -1.3 | 0.004 |
| rs41291187 | H624R | 29 | benign | 0.005 | tolerated | 0.33 | -1.7 | 0.002 |
| rs45608038 | N1787D | 23 | benign | 0.002 | tolerated | 0.93 | -3.6 | 0.001 |
| rs35062203 | N2945K | 94 | benign | 0.006 | tolerated | 0.3 | -0.7 | 0.085 |
| rs142278066 | H3881Y | 83 | benign | 0.064 | tolerated | 0.1 | 0.9 | 10.14 |

**Supplemental Table S3** A summary of scores obtained in assessing the missense variants in ALMS1 using the CADD (Combined Annotation Dependent Depletion) Tool v1.3. Scaled CADD scores of greater than 20 have been suggested to be indicative of possible loss-of-function variants.
