# Supplementary material for: Refining genotype–phenotype correlation in Alström syndrome through study of primary human fibroblasts
Source: Mol Genet Genomic Med. 2017 May 15;5(4):390–404. doi: 10.1002/mgg3.296 (PMC5511801; doi:10.1002/mgg3.296)
Supplement: Supplementary file 5 — Appendix S1. Supplementary Materials and Methods. [file MGG3-5-390-s005.docx]

**Refining Genotype-Phenotype Correlation in Alström Syndrome Through Study of Primary Human Fibroblasts**

**Chen et al**

**SUPPLEMENTARY MATERIAL**

**Supplementary Materials and Methods**

**Quantification of ciliary length and ALMS1 staining intensity**

Cilia and ALMS1 in the basal body were stained, respectively, with anti-acetylated tubulin or anti-ALMS1 antibodies as described. Ciliary lengths and ALMS1 staining intensity were measured using LAS AF Lite (Version 2.6.3 build 8173, Leica Microsystems CMS GmbH).

**TaqMan assay of ALMS1 mRNA expression**

Total RNA was extracted from replicating fibroblasts using RNeasy Mini Kits (74106, Qiagen) and first strand cDNA was reverse-transcribed from 800 ng of total RNA using an ImProm-II Reverse Transcription System (A3800, Promega) with random hexamers as the primer, according to the manufacturer’s protocol. Relative expression was determined with TaqMan assay using ALMS1 TaqMan primers/probe spanning exon 13 and exon 14 (Hs00367316_m1, Cat# 4331182, Thermo Fisher) with HPRT1 (Hs99999909_m1, Cat# 4331182, Thermo Fisher) as a “housekeeping” control.

**Mutation sequencing of cDNA from dermal fibroblasts of P7, P8, P10 and P21**

Total cellular RNA was prepared using RNeasy Mini Kits (Qiagen) and was quantified spectrophotometrically on a NanoDrop ND-1000 (Thermo Scientific). First strand cDNA was reverse-transcribed from 800 ng of total RNA using an ImProm-II Reverse Transcription System (A3800, Promega) with random hexamers as the primer, according to the manufacturer’s protocol. cDNA was then used for PCR using primers encompassing mutation sites of each patient (for P7: forward primer, CACACTCGAGATGTTGGGAT; reverse primer, CTGAAGGGTTGCTCTCACAA; for P8: forward primer, GAAAACCATTCTCCCCTTCC, reverse primer, GAGGTTGGTTCCAGTGATCT; for P10: forward primer, AGCATTTTCTACCAGCAGGG; reverse primer, AGAGAGTACTGTTGCTGTCC; for P21 (p.Trp265*): forward primer, CAAGTAAAGGAACCCAACAGA; reverse primer, AACGATCACAGTCTTTGGGA; P21 (p.Arg3701*): ACACATTCTCTCCAGGTCTC; reverse primer, GCATACTCTTTCATGGCCAA). PCR products were Exo1/SAP treated and then used for sequencing reaction using BigDye terminator (4336919, Applied Biosystems) according to the manufacturer’s protocol. Primers used for sequencing reaction are as follows: P7, GAGAACAGTGATGTGACTTC; P8, CCGTCTCCCATTTCTCTTGA; P10, CTTGCCAGACAGTCATCTAA; P21 (p.Trp265*), CCTTTGCTGACCTGTTTGAC; P21 (p.Arg3701*), GAATGGAGTGGTAGACAACA. Sequencing extension products were purified using BigDye cleaning beads (BCB-100, MCLAB) and then analysed with an ABI3730 DNA analyser. DNA sequence data were analysed with Sequencher software (Gene Codes Corporation).
